# Joint Preparation and Ray Shortening in Arthroscopic Versus Open First Metatarsophalangeal Fusion: A Cadaver Study

**DOI:** 10.7759/cureus.9633

**Published:** 2020-08-09

**Authors:** Haley McKissack, Bradley Alexander, Gean C Viner, Eildar Abyar, Nicholas A Andrews, Ashish Shah

**Affiliations:** 1 Orthopaedic Surgery, University of Alabama at Birmingham, Birmingham, USA

**Keywords:** mtp fusion, arthroscopic, joint preparation, ray shortening

## Abstract

Purpose

This study compares the amount of joint preparation and first ray shortening following first metatarsophalangeal (MTP) joint fusion utilizing open conical reaming versus arthroscopic technique.

Methods

Ten below-knee cadaver specimens were randomly assigned to undergo either open or arthroscopic first MTP fusion. Following fixation, first ray length measurements were obtained from pre-operative and post-operative radiographs and were used to determine first ray shortening. Additionally, the ratio of first ray length to second ray length was calculated both pre-operatively and post-operatively and compared between the two approaches. All ankles were then completely dissected, and prepared surface areas were demarcated. ImageJ photo analysis software (National Institutes of Health, Bethesda, MD, USA) was used to calculate the percentage of prepared and unprepared cartilage of each articular surface of each specimen.

Results

Overall, the open approach resulted in 99.3% ± 1.6% joint surface preparation, whereas the arthroscopic approach yielded 92.9% ± 7.2% (p = 0.089). On average, the head of the first metatarsal was significantly more prepared with the use of the open approach (99.5% ± 1.1%) than with the arthroscopic approach (96.6% ± 1.5%) (p = 0.008). However, with respect to the base of the phalanx, the average difference in preparation between the arthroscopic approach and the open approach was not statistically significant (90.0% ± 12.8% vs. 99.0% ± 2.2%; p = 0.160). The average amount of first ray shortening in the arthroscopic approach was 2.2 ± 1.8 mm compared to 2.1 ± 3.2 mm in the open approach (p = 0.934). The average change in the first to second ray length ratio was 0.02 for both approaches (p = 0.891).

Conclusion

Arthroscopic first MTP fusion can be used to achieve joint preparation comparable to open technique while maintaining first ray length.

## Introduction

Open and arthroscopic first metatarsophalangeal (MTP) joint fusions are effective treatments for a variety of conditions such as rheumatoid arthritis, hallux rigidus, and severe hallux valgus [1]. Multiple surgical techniques have been described in the literature with regard to bone preparation and joint fixation, with varying degrees of success [2-6]. Regardless of technique, the important aspects of fusion are adequate preparation of the joint, maintaining the soft tissue envelope, and stable fixation. Singh et al. compared first MTP joint fusion utilizing flat cut or conical reaming technique for bone preparation with regard to the amount of first ray shortening that occurs and found no significant difference between both techniques [4]. These two techniques require an open approach to access the joint for surface preparation. In contrast, the arthroscopic technique is a minimally invasive procedure that allows joint visualization and surface preparation with less soft tissue damage. The first study describing first MTP arthroscopy was published in 1972, but due to a number of factors, arthroscopic treatments for first MTP joint have not fully spread [7,8]. This could be attributed to the learning curve that comes with arthroscopic surgery and the proven success of the open approach in first MTP fusion.

One potential complication of first MTP fusion is first ray shortening, which can lead to symptomatic forefoot disorders such as transfer metatarsalgia of the lesser toes [6,7,9]. The second metatarsal seems to be especially vulnerable to developing transfer metatarsalgia due to it being usually longer than the other metatarsals and fixed between three cuneiforms, making it relatively immobile [10]. Patients can develop altered gait mechanics that manifest in the way of decreased ankle plantarflexion at toe-off and decreased step gait [11]. Currently, there is no consensus regarding the amount of first ray shortening that is acceptable [9]. To our knowledge, no study has compared the difference in joint preparation or the amount of first ray shortening following first MTP joint fusion utilizing open versus arthroscopic technique. The purpose of this study was to compare first MTP joint fusion utilizing open conical reaming versus arthroscopic technique for joint preparation with regard to the amount of first ray shortening that occurs. We hypothesize that arthroscopic MTP fusion will be as effective as the open technique, without any increase in first ray shortening.

## Materials and methods

Specimen preparation

Ten fresh-frozen, unmatched below-knee cadaver leg specimens were utilized. Each specimen was inspected visually and radiographically to ensure the absence of any gross musculoskeletal pathology. Due to the nature of these specimens, we were not able to review or compare past medical records. There were four male donors and six female donors. Mean specimen age at death was 66.2 ± 15.3 years (range: 36-86 years). All specimens were stored at -20°C and then thawed at room temperature for 24 hours prior to testing.

Open technique

All specimens were prepared by a single foot and ankle fellowship-trained orthopedic surgeon. The first MTP joint was approached through a standard dorsal longitudinal incision. Soft tissue was dissected and extensor hallucis longus was freed and carefully retracted. The dorsal aspect of the joint capsule was incised and elevated subperiosteally over the first metatarsal and proximal phalanx to allow exposure and preparation of the joint. A 1.6-mm Kirschner (K)-wire was inserted into the metatarsal head, and a conical reamer (Arthrex Inc., Naples, FL, USA) was slid over the guidewire to expose the subchondral bone. The proximal phalanx was then prepared in a similar fashion. The joint was then placed in 15 degrees of dorsiflexion and 10 degrees of valgus using a 1.6-mm K-wire and a compression screw placed from distal-medial to proximal-lateral in a standard lag fashion. Anterior-posterior and lateral fluoroscopic images were obtained following fixation.

Arthroscopic technique 

All specimens were prepared by a single foot and ankle fellowship-trained orthopedic surgeon. The great toe was distracted with traction over a pulley attached to the surgical table. A 30-degree, 2.4-mm arthroscope (Arthrex Inc.) was used for visualization. A two-portal approach was utilized in five feet. The dorsomedial portal site was marked at the medial aspect of the first MTP joint line, and the dorsolateral portal site was marked on the lateral aspect of the extensor hallucis longus tendon at the level of the first MTP joint line. An 18-gauge needle was inserted at the marked site, and the skin incision was widened utilizing a hemostat. The arthroscope was inserted into one portal, and a 2.4-mm arthroscopic shaver (Arthrex Inc.) was inserted into the other. The shaver was used to remove the articular cartilage and expose the subchondral bone. The dorsolateral and dorsomedial portals were used interchangeably for working and viewing until both joint surfaces were adequately prepared. Following joint preparation, the MTP joint was then fixed in 15 degrees of dorsiflexion and 10 degrees of valgus using a 1.6-mm K-wire. The MTP joint was then fixed with a compression screw placed from distal-medial to proximal-lateral in a standard lag fashion. Anterior-posterior and lateral fluoroscopic images were obtained following fixation.

First ray length measurement

**Figure 1:**
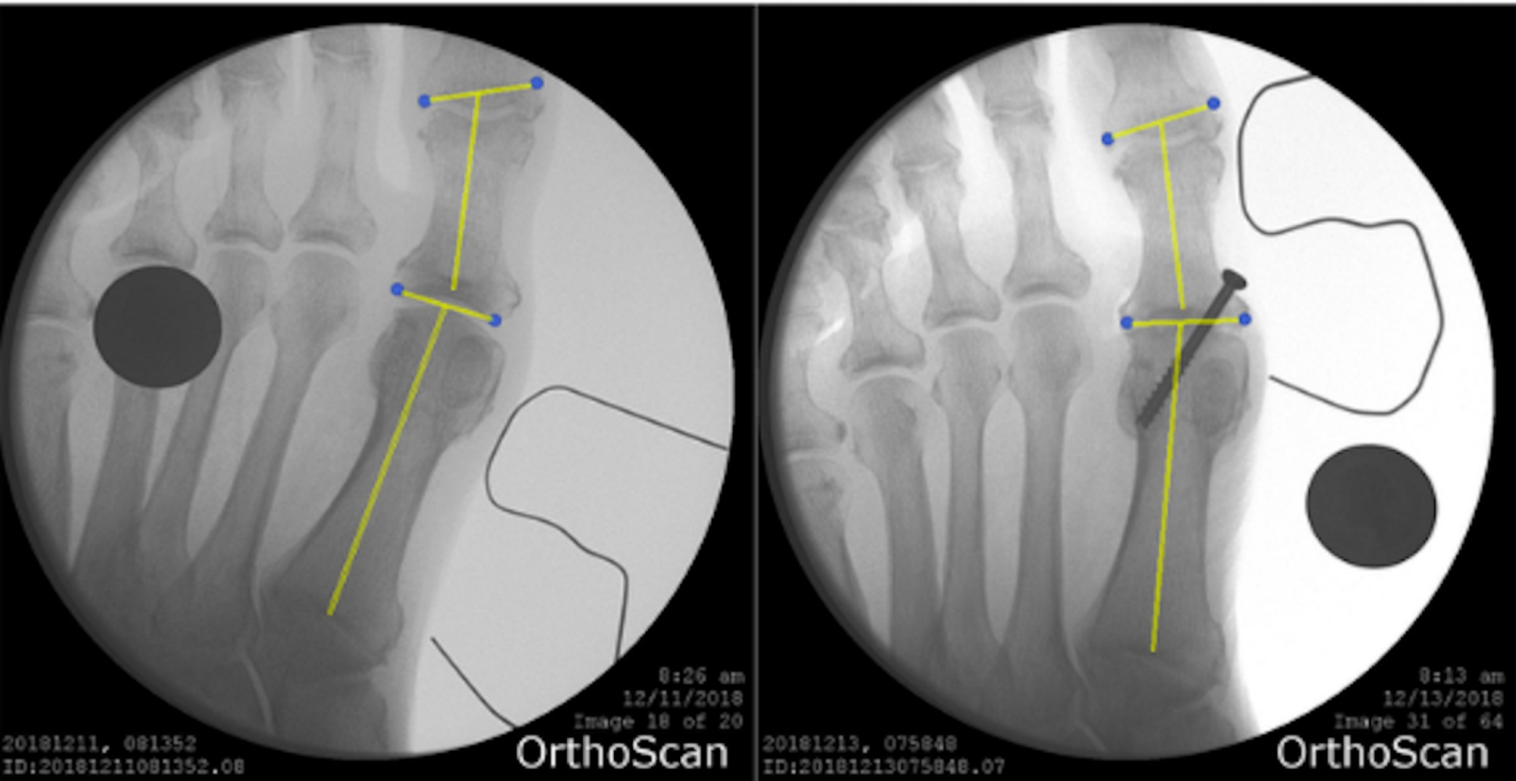
First Ray Plain Film Pre- and post-operative measurement of the first ray length using ImageJ software analysis of fluoroscopic images.

Pre-operative and post-operative lengths of the first metatarsal were measured from anterior-posterior radiographs using ImageJ (Wayne Rasband, National Institutes of Health, Bethesda, MD) (Figure 1). The length of the first ray was measured from the base of the first metatarsal to the joint line of the head of the first proximal phalanx, as described by Singh et al [4]. The line of measurement was drawn from the midpoint of the joint line of the first metatarsal base to the midpoint of the joint line of the proximal phalanx head. This technique is demonstrated in Figure 1. Similarly, length of the second ray was measured from the base of the second metatarsal to the distal end of the head of the second proximal phalanx. Change in pre-operative and post-operative length of the first ray was calculated for each specimen. The average change in first ray length was compared between specimens prepared using the arthroscopic approach and those prepared using the open approach. Additionally, the ratio of first ray length to second ray length was calculated both pre-operatively and post-operatively and compared between the two approaches. 

Joint preparation measurement

**Figure 2:**
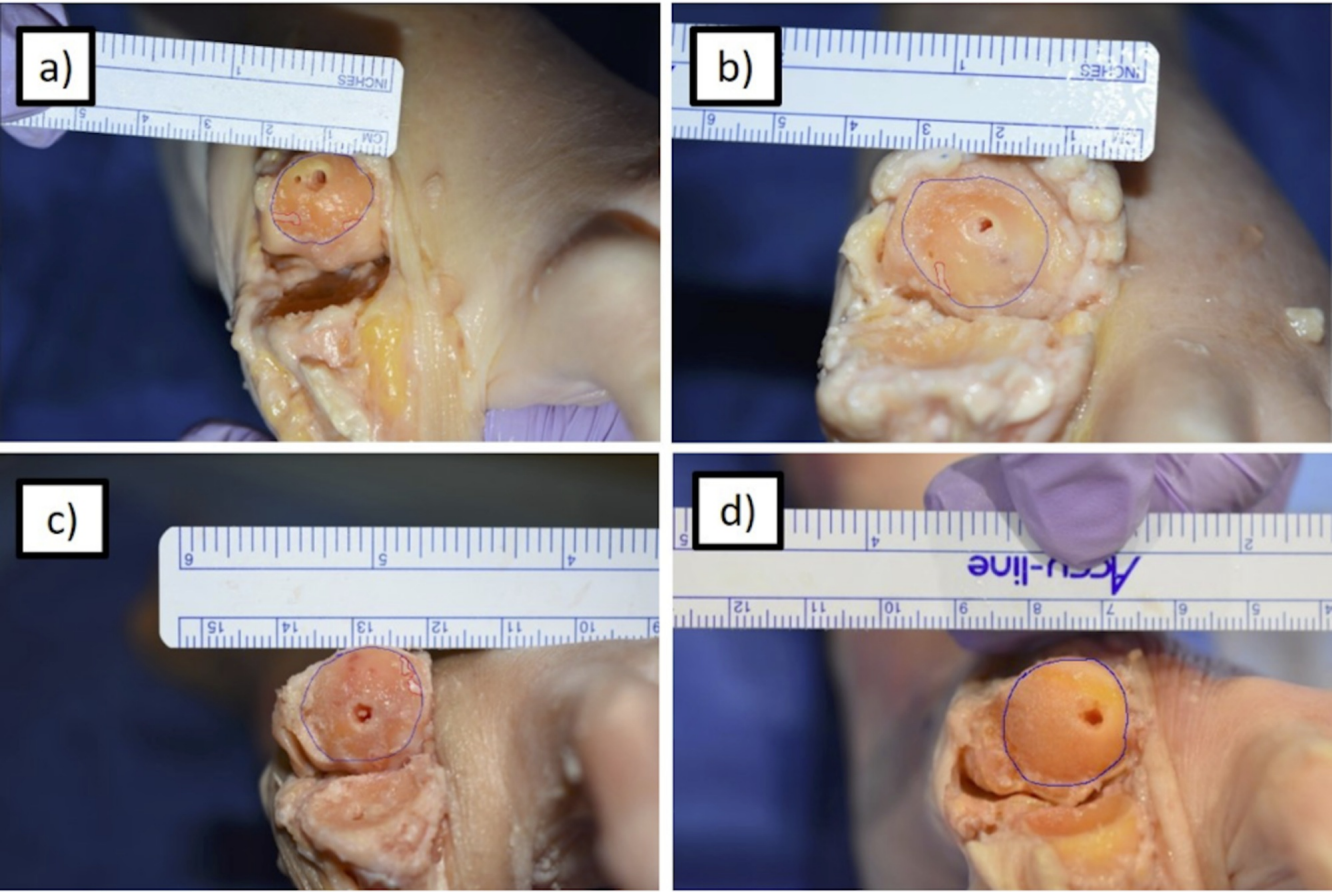
MT Head Preparation Measurement of total prepared fusion contact surface area using ImageJ software for (a) the most incomplete arthroscopically prepared MT head, (b) the most complete arthroscopically prepared MT head, (c) the most incomplete open technique prepared MT head, and (d) the most complete open technique prepared MT head. The blue circle represents the estimated fusion contact surface area. Red outlines represent areas of remaining articular cartilage. MT, metatarsal

Following completion of arthrodesis and after fluoroscopic images were obtained, the joint was dissected to expose the articular surface of each side of the MTP joint. High-resolution pictorial images of both joint surfaces were obtained. Estimated fusion contact area was assessed and outlined, and the total fusion contact surface area and the amount of unprepared cartilage on the proximal phalanx and distal metatarsal head were measured using ImageJ software (Figure 2) [12-14]. These measurements were used to calculate the percentages of prepared and unprepared cartilage of each articular surface for each specimen.

Statistical analysis

Data were collected and analyzed using descriptive statistics in Microsoft Excel (Microsoft Corp., Redmond, WA, USA) and SPSS Statistics (IBM, Armonk, NY, USA). All outcome measures were compared using paired t-tests to examine the difference between the arthroscopic and open MTP fusion technique groups (α ≤ 0.05). An a priori power analysis was performed to determine what power could be achieved based on the number of specimens available for this investigation. Based on the clinical experience of board-certified foot and ankle orthopedic surgeons, it was determined that a 10% difference in joint preparation and 5-mm difference in ray shortening would be clinically significant. Four specimens were in each group for ray shortening analysis yielding a beta of 0.26, and five specimens were in each group for joint preparation analysis yielding a beta of 0.72.

## Results

Prepared surface area

The percent of joint preparation for each cadaver is detailed in Table 1. On average, the head of the first metatarsal was significantly more prepared with the use of the open approach (99.5% ± 1.1%) than with the arthroscopic approach (96.6% ± 1.5%) (p = 0.008). However, with respect to the base of the phalanx, the average difference in preparation between the open approach and the arthroscopic approach was not statistically significant (99.0% ± 1.6% vs. 90.0% ± 12.8%; p = 0.159). When assessing the average total prepared surface area of the entire joint with both articular surfaces combined, the open approach yielded an average of 99.3% ± 1.6%, whereas the arthroscopic approach yielded an average of 92.9% ± 7.2. These differences were not statistically significant (p = 0.089).

**Table 1:** Percent of Joint Surface Prepared by Cadaver

Cadaver No.	Preparation Method	Percent of Metatarsal Prepared	Percent of Phalanx Prepared	Percent of Total Joint Surface Prepared
1	Scope	95.2	69.7	80.8
2	Scope	98.5	85.8	91.3
3	Scope	96.1	100.0	98.1
4	Scope	95.5	100.0	97.9
5	Scope	98.0	94.6	96.1
6	Open	100.0	100.0	100.0
7	Open	100.0	100.0	100.0
8	Open	97.6	95.0	96.4
9	Open	100.0	100.0	100.0
10	Open	100.0	100.0	100.0

First ray length

Radiographs for eight total specimens, four in the arthroscopic group and four in the open group, were available for first ray length analysis (Table 2). Average decrease in length of the first ray using the open approach was 2.1 ± 3.2 mm, whereas average decrease using the arthroscopic approach was 2.2 ± 1.8 mm. The difference in the average change in length between the two approaches was not statistically significant (p = 0.934). The average change in the first to second ray length ratio was 0.02 for both approaches (p = 0.891). Of note, the length of one specimen prepared using the open approach increased by 1 mm, presumably secondary to correction of preoperative hallux valgus. We did not see any differences in change in ray length based on the gender or age of the cadaver specimen.

**Table 2:** MTP Shortening by Cadaver MTP, metatarsophalangeal

Cadaver No.	Preparation Method	Ratio Difference	Length Difference (mm)
1	Scope	0.04	4.267
2	Scope	Radiograph unavailable	
3	Scope	0.01	0.791
4	Scope	0.01	0.77
5	Scope	0.03	3.162
6	Open	0.06	6.37
7	Open	Radiograph unavailable	
8	Open	-0.01	-1.01
9	Open	0.03	2.64
10	Open	0.00	0.36

## Discussion

First MTP fusion has been shown to improve the stability, propulsive power, and overall function of the foot, with high patient satisfaction rates [15-17]. The success rate of first MTP fusion was shown to be 90% in one study [18]. More recent studies have shown that revision rates for first MTP fusion are between 0% and 11.7% [19]. One of the complications of first MTP fusion is transfer metatarsalgia due to shortening of the first metatarsal. Jung et al. demonstrated an increase in plantar pressure of the second to fifth metatarsals when shortening of the first metatarsal occurs [9]. The increase in pressure was found to be proportional to the increase in first ray shortening. Additionally, the first MTP plays an important role in normal gait by bearing 40% to 60% of body weight, which increases two to three times during running activities. Therefore, first ray length must be maintained during joint preparation for MTP arthrodesis. Singh et al. investigated the amount of first ray shortening in first MTP arthrodesis using flat cuts compared to conical reamers and concluded that there was no significant difference in terms of shortening [4]. The results of our study demonstrate no significant difference in the amount of first ray shortening between arthroscopic and open joint preparation.

As with any fusion, outcomes of first MTP fusion are dependent on adequate preparation of the joint, regardless of surgical technique. For first MTP fusion, joint preparation can be accomplished through an open approach using a conical reamer or flat cutter or with an arthroscopic technique using an arthroscopic shaver. To our knowledge, this is the first study to compare joint preparation and first ray shortening between an open conical reaming technique and arthroscopic technique. In our study, the difference in the amount of overall joint preparation between the open and arthroscopic groups was not statistically significant. Although the difference in the amount of total surface area prepared on the metatarsal head between the two groups was statistically significant, it is unclear whether this difference is clinically relevant. The percent of the metatarsal head that was prepared using arthroscopy in our study was similar to the amount prepared in a study by Vaseenon and Phisitkul utilizing a similar arthroscopic technique (92.9% vs. 93.31%) [20].

In general, arthroscopic technique is associated with decreased infection rate, scarring, and bleeding, as well as improved cosmesis and faster recovery [21]. MTP arthroscopy was first described by Watanabe in 1972 [8]. In recent years, the role of arthroscopy of the first ray has expanded as it has been described in the treatment of various forefoot pathologies [22]. Ahn et al. described a series of 59 patients with a variety of pathologies treated with first MTP arthroscopy that demonstrated an improvement in outcomes scores, high patient satisfaction with the procedure, and a low rate of complications [23]. Additionally, Lui reported on 121 cases of first MTP arthroscopies in patients with hallux valgus and found that of patients with preoperative MTP joint pain, 90% had complete or significant relief of pain postoperatively [24]. Similarly, arthroscopic ankle arthrodesis has been shown to result in better long-term outcomes scores and shorter hospitalization [25].

Although arthroscopic management of forefoot pathologies has several advantages, it is not without limitations. Arthroscopy of the forefoot is technically demanding and requires adequate training and surgical experience. First MTP arthroscopy is most commonly performed through two dorsally based portals, which lead to difficulty visualizing and treating dorsal structures. Although our study utilized two portals, a three portal technique has been shown to provide better exposure and allow more joint preparation when compared to two-portal technique [20]. Similarly, newer techniques have been described utilizing four portals for increased visualization of the MTP joint. Additionally, arthroscopy may not be possible in patients with high angular deformity or those with severe arthritic changes due to osteophyte formation and lack of joint space. Furthermore, traction is necessary for performing arthroscopy in order to widen the joint space, which may result in nerve injury.

Valuable information can be gained from a study that uses a cadaveric model. However, there are limitations on the number of specimens that can be acquired for these types of studies. Despite the power being inadequate due to the logistical limitations of having access to more specimens, the study is still the first of its kind to look at how surgical technique effects ray shortening and joint preparation in MTP fusion, therefore still providing value to the field of foot and ankle surgery. Second, the specimens used for this study did not include severely arthritic joints, thus it is difficult to assess how effective arthroscopic joint preparation is in patients with significant MTP joint pathology. Finally, we used anterior-posterior simulated weight-bearing fluoroscopic images for measurements and it is possible that the axis of the beam did not align with the anterior-posterior axis of the foot. We attempted to minimize this error by measuring the length of the second metatarsal and including the ratio of first and second ray lengths in the analysis. Finally, the accuracy of our measurement is limited by the ImageJ software; however, the same software was used on all specimens, and the effect of this error is felt to be minimal.

## Conclusions

Arthroscopic first MTP fusion can be used to achieve comparable total joint preparation to open technique despite increased metatarsal head preparation in the open group. The clinical significance of increased metatarsal head preparation is currently unknown. First ray length was maintained in both arthroscopic and open groups. Our study is the first to examine how surgical technique affects ray shortening and joint preparation in MTP fusion. This cadaveric study’s findings suggest that arthroscopic first MTP fusion is likely an effective surgical option for surgeons with adequate arthroscopic experience. Further research is necessary to correlate our findings clinically.
